# Automatising the analysis of stochastic biochemical time-series

**DOI:** 10.1186/1471-2105-16-S9-S8

**Published:** 2015-06-01

**Authors:** Giulio Caravagna, Luca De Sano, Marco Antoniotti

**Affiliations:** 1 Dipartimento di Informatica, Sistemistica e Comunicazione, Università degli Studi di Milano-Bicocca, Viale Sarca 336, I-20126 Milan, Italy

**Keywords:** time-series analysis, stochastic models, Python

## Abstract

**Background:**

Mathematical and computational modelling of biochemical systems has seen a lot of effort devoted to the definition and implementation of high-performance mechanistic simulation frameworks. Within these frameworks it is possible to analyse complex models under a variety of configurations, eventually selecting the best setting of, e.g., parameters for a target system.

**Motivation:**

This operational pipeline relies on the ability to interpret the predictions of a model, often represented as simulation time-series. Thus, an efficient data analysis pipeline is crucial to automatise time-series analyses, bearing in mind that errors in this phase might mislead the modeller's conclusions.

**Results:**

For this reason we have developed an intuitive framework-independent Python tool to automate analyses common to a variety of modelling approaches. These include assessment of useful non-trivial statistics for simulation ensembles, e.g., estimation of master equations. Intuitive and domain-independent batch scripts will allow the researcher to automatically prepare reports, thus speeding up the usual model-definition, testing and refinement pipeline.

## Background

The study of biological systems witnessed a prominent cross-fertilisation between experimental investigation and computational methods, thanks to many different modelling approaches developed within different research areas (e.g, biophysics, computational biology, logics) to describe natural systems [1]. Nowadays, many such studies can be ascribed to the area of *Systems Biology*, an approach characterised by studying complex interactions in natural systems and their properties as wholes, not as collections of parts [2,3]. This holistic discipline requires us hence to consider a system at multiple simultaneous abstraction levels, e.g., from RNA to proteins up to abundant chemical signals.

In mathematical sciences various theoretical frameworks for mechanistic modelling, which can be ascribed to the broad categories of *mean-field equations *or *stochastic processes*, have been introduced to capture the *physics *underlying a target system, at some level of abstraction. These frameworks provide fundamental ingredients to achieve successful results in Systems Biology. These ingredients are, for instance, the ability to naturally describe *multi-scale *interactions and components by combining different mathematical frameworks (more often termed *hybrid*) in a sort of orthogonal approach [4,5].

In the specific context of natural systems, another key feature of successful frameworks is the ability to consider *stochasticity*. This, meant either as the random fluctuations intrinsic to the model components present in few copies or as the fluctuations induced by extrinsic sources, is recognised to have a fundamental role in many living processes [6,7]. Stochastic phenomena arise, for instance, in the bursts of protein transcription (molecular level), in cell-fate decision processes of differentiation (cellular level) and in evolutionary transitions (population level) [8-13]. Thus, interest has increased towards modelling frameworks which support one or more forms of stochasticity, e.g., Markovian Gillespie-like approaches, stochastic differential equations or non-Markovian hybrid automata to name but a few [14-21].

In general, regardless of the way in which stochasticity is embedded in a framework, many analyses rely on the evaluation of *simulation ensembles*, under different model configurations and parameters. Then, an efficient data analysis pipeline is set up to produce reports of the model predictions usually in the form of, e.g., *probability measures *which emerge "naturally" by the intrinsic random nature of the model itself. Evaluating such quantities sometimes requires a noteworthy computing time, a price that we need to pay to assess whether a model reaches an *equilibrium behaviour*, or if *stochastic bifurcations*, *resonances *or *multi-stable phenomena *arise.

All in all, according to the physics of the system under study (e.g., number of components, the type of interactions, and thermodynamic setting) a suitable modelling framework can be chosen and, quite likely, a simulation tool can be found in the literature. Sometimes these are coded within pre-packaged scientific tools such as Matlab, R or Mathematica. However, these general-purpose frameworks offer many functions beyond the scope of plain simulation, and thus one often codes tools in a lower-level programming environment to boost performance, a key issue [22-27].

Historically most of the efforts have been devoted to the definition of high-performance simulation frameworks, while less has been done to automate the inherently *time-consuming *and definitely *error-prone *task of data analysis. Thus, adhoc analysis tools are often implemented to process the output of a variety of simulators/experiments and produce the required statistics. Obviously, when this pipeline is either inefficient or incorrectly implemented hours of computation-intensive calculations can be lost, and a whole research activity slowed down. Of course, the general-purpose tools mentioned above, e.g., Matlab, R or Mathematica can be used to perform analyses of time-course simulation results. However, they require extra learning time to master and, in general, provide many complex functions actually unused for this purpose.

### PYTSA: a Python Time-Series Analyser

**Figure 1:**
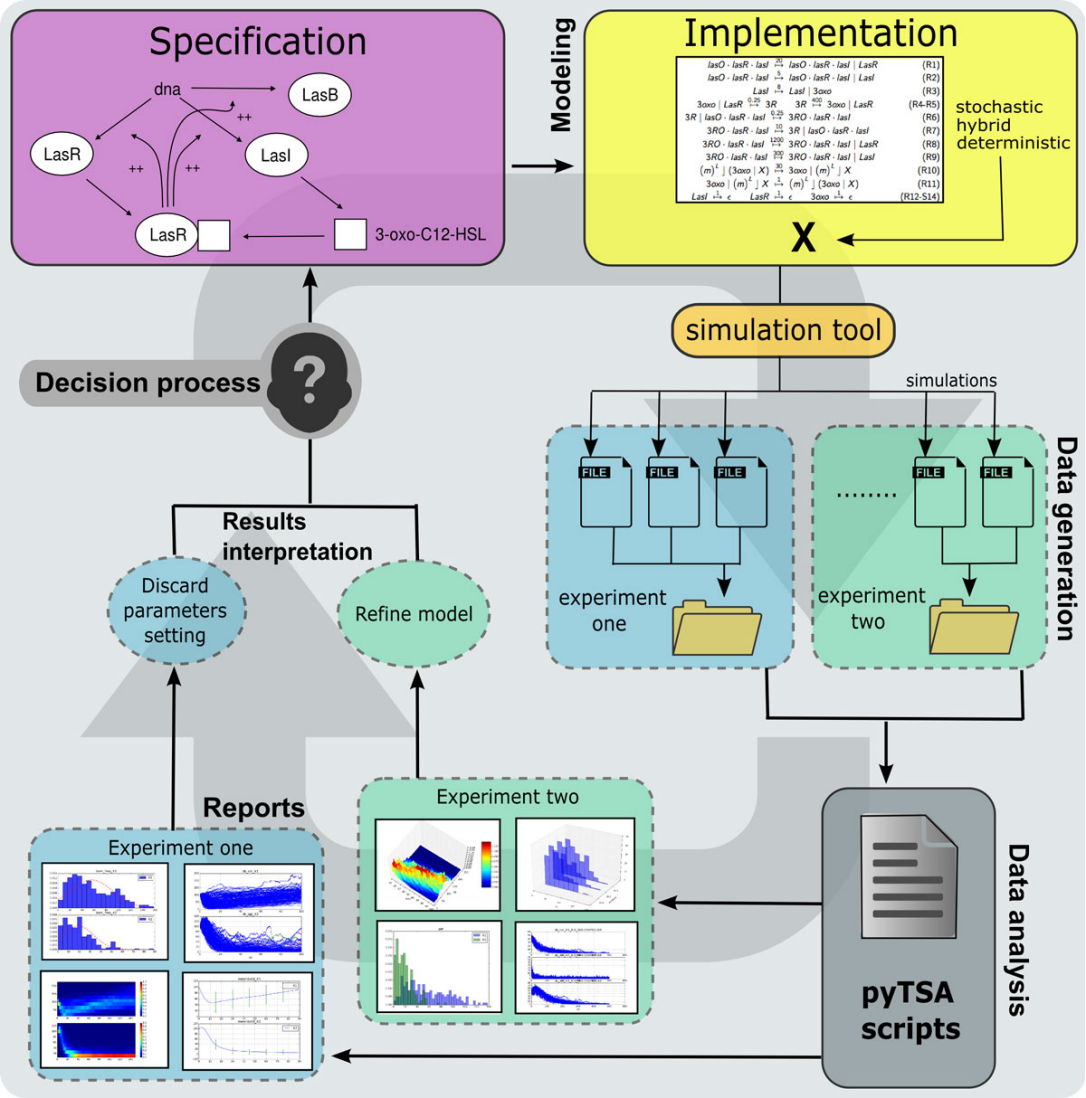
**PYTSA pipeline for automatic data analysis**. PYTSA generates reports of simulations by analysing output time-series. Input data is expected to be obtained by running simulations of a model, which is implemented from some specification and is simulated by some tool. Output time-series of simulations should be stored in some folder, and a PYTSA script should be prepared to perform the desired analyses (see Table 1 for available PYTSA commands). By executing the script a report of the model predictions can be generated (possibly for various independent sets of simulations, denoted as "experiments" in the figure) and a decision process can be started to either accept or refine the model, or any of its constituting components.

We developed a Python *Time-Series Analyzer *(PYTSA, pronounced as "pizza") as a first step to collect into a lightweight, simulation-framework-independent and focused library a number of typical routines (and relative scripts) to analyse synthetic/wet biological time-course simulation experiments. PYTSA is flexible in the way several analyses can be easily combined, with intuitive commands, in a pipeline with any simulation tool outputting time-series, thus providing a modeller with more options to produce a report from his work (see Figure 1). The wide diffusion of Python for scientific computing makes this library embeddable in other analysis frameworks and, also, PYTSA's input/output support for the *Systems Biology Results Markup Language *(SBRML, [28]) allows one to load and process results obtained with most of the widely available simulation tools.

The current PYTSA version introduces the notion of *dataset *as a set of files in some folder, and assumes that each file represents a independent time-evolution of a model. To boost loading of huge datasets, a multi-process implementation is adopted. In each data file, columns represent the values of a model variable to which a mnemonic name can be assigned, for clarity (see the Example below). Notice that, in principle, this input format is domain-independent, thus non-biological data can be analysed as well. The library supports standard plotting routines and output formats, which either manipulate or not data. In the former case one might just visualise data as, e.g., *single traces *or 2D/3D *phase-spaces *for some time window or model variable. In the latter case one could estimate averages, standard deviations, or probabilities of any variable. Note that *probability densities *of a variable either at some specific time point, or over a time interval, provide an estimation of the (solution of the) *master equation *for the model.

This equation, central to stochastic models, see e.g. [14], describes the probability of observing a configuration of the model over time and, in general, its solution is assessable solely via numerical simulation ensembles. If, for example, one were to model a gene regulatory network, questions such as *"what is the probability of having a certain RNA concentration after some time units of network activity" *could be answered by using this equation - in the Supplementary Material we show a simple example of its estimation for the case study described in the main text.

PYTSA plots these statistics with multi-panel visualisation, barplots, heatmaps, 3D surfaces, normalisation and gaussian-fit. All in all, despite the statistics implemented in PYTSA being rather intuitive, we believe that researchers would benefit from a tool specifically tailored to automatise, in a easy fashion and with a domain-specific language, data analysis of stochastic time-traces. Hopefully, this should eventually allow the researcher to focus on research tasks beyond data analyses, thus speeding up the usual pipeline of model-definition, testing and refinement.

Finally, efforts towards a more standardised definition of what a "simulation" result are under way. For instance, the SBML community has been working on several standards that aim to connect models, datasets and simulations (cfr., SED-ML [29], SBRML and Teddy [30]). PYTSA fits into these frameworks by already supporting SBRML in its current version, and it will be further developed to adhere to other standards. To conclude, PYTSA was conceived to analyse systems where the variation of concentration of some molecules is reported, without spatial information. Spatial models which describe, e.g., tissues and organs, have usually more complex output formats according to the notion of "space" they implement, e.g. [24,31-35]. If proved successful, our tool could be extended to support spatial outputs in the next versions.

## Implementation

PYTSA sources are available (see [36]) and the key implemented functions are summarised in Table 1. The manual of the version leads the reader to a wizard-like understanding of PYTSA. The library relies on some standard Python libraries to delegate the heaviest computations: NUMPY [37], SCIPY [38], MATPLOTLIB [39], Pandas [40] and PyTables [41] which provide functions to either manipulate mathematical objects or process input/output data.

**Table 1:** Data-processing PYTSA functions

Function	Synopsis
timeseries	*load a time-series from a single file (output of a single simulation) *

dataset	*load a dataset of time-series (output of repeated simulations) *

splot	*plot plain time-series (without any processing) *

aplot, sdplot, asdplot	*plot average, standard-deviation and both of them for a dataset *

phspace2d, phspace3d	*plot 2D/3D phase-spaces for plain time-series *

aphspace2d, aphspace3d	*plot 2D/3D phase-spaces for the average of a dataset *

pdf, pdf3d	*plot the probability density of a model variable at one or more (3D) time-points (requires a dataset) *

meq2d, meq3d	*estimates the master equation solution for a model variable in the form of time-varying probability density for a time-interval (heatmap 2D or surface 3D, requires a dataset) *

**Figure 2:**
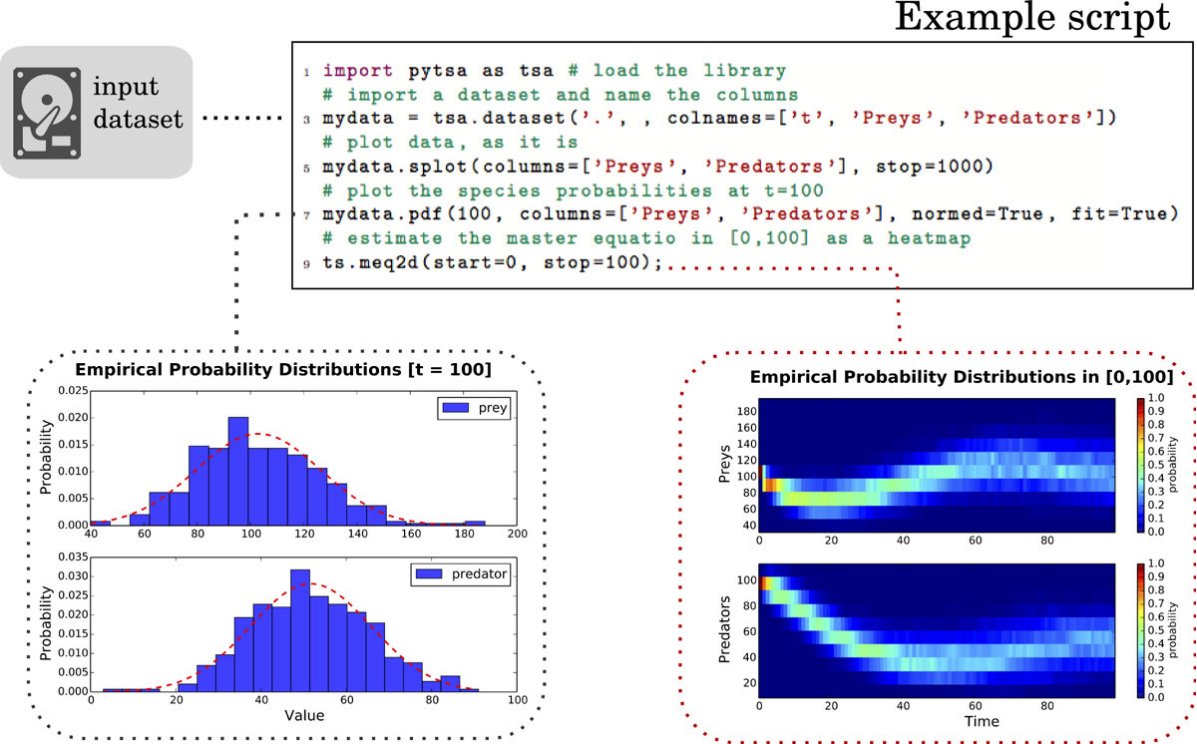
**Example report for the prey-predator model**. PYTSA example report produced for a dataset of 100 independent simulations of a prey-predator model realised with NOISYSIM [26]. Dataset and the analysis script are available at [36]. The above script loads data where each output file is expected to have at least three columns (*t *stands for time, the free variable). Then, all the loaded time-series are visualised without processing for *t *≤ 1000, and the probability distribution of preys/predators is numerically estimated (with normalisation an Gaussian fit) at *t *= 100. Finally, in the interval $t\in \left[0,100\right]$ the time-varying probability distribution is evaluated too, yielding a heatmap representation of the approximate solution of the master equation ruling the dynamics of preys/predators. This allows to investigate, visually, the model stability and possibly raise further questions about the species behaviour.

These functions (plus others unreported here which allow the user to customise the running environment) are natively implemented within PYTSA and can be combined in scripts for batch processing, or interpreted in any Python interactive environment. Each of these functions has a complex set of input parameters, whose meaning and usage is documented in the tool manual, where examples are provided, see [36]. A simple explanatory script which makes use of some of these commands is reported and commented in Figure 2.

The PYTSA implementation consists of 4 classes (see the technical specifications at [36]). A fork-based implementation ensures an optimal parallel data-loading based on the number of available cores, and a cache-based implementation avoids the repetition of a computation already performed on some data, to optimise speed. Preliminary testing shows that on a 4-core machine parallel data-loading is 300% faster than with a sequential one. Also, loading of datasets of size exceeding 10 GB seems prohibitively slow without a parallel implementation. Further memory-optimisation is given by NUMPY and PANDAS to efficiently store arrays and time-series, meanwhile processing data. Also, statistical routines provided by SCIPY allow one to efficiently evaluate fits of histograms and binning (i.e., a form of quantisation used to aggregate values which fall in a given small interval, a bin, to obtain a value representative of that interval) and, finally, by using MATPLOTLIB, advanced plot-editing capabilities are possible (i.e., a visualised plot can be modified by using the underlying PYTSA tool and its imported libraries, allowing the advanced user to benefit from the power of all the tools interfaced with PYTSA).

## Results

We denote with **X** the model of a system under study, whose time-evolution can be either deterministic, stochastic or hybrid (see Figure 1). The point is to gather information from a dataset of realisations of **X** which describe its variables in the form of a time-trace. These datasets are often stored to disks and processed offline, and sometimes several hours are required to set up the analysis framework, load, process and interpret data. This process, very sensitive to errors, is eased by PYTSA.

Aside from plain traces visualisation, numerical statistics can be evaluated:

• the *average *$\left\langle \text{X}t\right\rangle$ of n traces, and its *standard deviation *$\sigma_{X}$;

• the probability density at a time t of X's variables, $P\left[\text{X}\left(t\right)=\text{X}\right]$, i.e. the probability of the system to be in a configuration × at time t;

• the above probability in a time interval. This, is analogous to estimating the solution of the *master equation *$\partial tP\left[\text{x},t\right]$ which rules the time-evolution of the probability of the system to be in some configuration × [42].

### Example report generation

The Lotka-Volterra predator-prey models are a family of models describing the dynamics of competitive populations living in an environment. These are based on the competition between species together with their evolution and, because of their generality, these equations are often used to model, e.g., *microbial population dynamics*. When one analyses these kind of models is often interested in finding parameter settings which guarantee the *environmental sustainability*, i.e. the situation in which the two species oscillate in time, without extinction.

Here we show a simple analysis of a dataset describing 100 independent simulations of the model realised with NOISYSIM [26]. In the Additional file 1 we provide details on the model specification, the parameters used for its simulation and other kind of questions which can be easily answered with PYTSA's analysis capabilities. To analyse stability of the ecosystem we use, as rough measure, the estimation of the time-varying probability of preys/predators in the time interval [0, 100]. We show how this can be easily assessed with the following 5-line PYTSA script:

1 import pytsa as tsa # load pyTSA

    # import a dataset and name the columns

3 mydata = tsa.dataset(".", colnames =["time","Preys","Predators"])

    # just visualize the loaded traces for t< = 1000

5 mydata.splot(columns =["Preys","Predators"], stop = 1000)

    # plot the species probabilities (normalized and fit) at t = 100

7 mydata.pdf(100, columns =["Preys","Predators"], normed=True, fit=True)

    # estimate the master equation in [0, 100], show it as a 2D heatmap

9 mydata.meq2d(start = 0, stop = 100)

Results of executing this script are sketched in Figure 2 (dataset and script are available at [36]); a more complete analysis is shown in Additional file 1.

Notice in that case the definition of the mnemonic names Preys and Predators (at data-loading time) and in the successive plot commands splot, pdf and meq2d which produce the plots shown in the Figure 2 (script processing time approx. 20 *sec*). When this report is generated further information is also returned within the Python environment, e.g., the minimum and maximum values for each species, the parameters µ and σ^2 ^of the Gaussian fit (as performed by the scipy library with a numerical algorithm which uses explicit formulas for the maximum likelihood estimation of the parameters).

The estimation of the solution of the master equation for this system allows the modeller to make inferences about stability of the populations. In particular, as it can be observed in the figure, for the parameters used to carry out the simulations and for the considered time-window the species oscillate with a period of about 80 days. Also, predators reach low values with non-negligible probability, i.e., observe the area where *Y*_2 _< 20, which might threaten predators' survival. With this in mind it is possible to raise and answer questions such as: *"What is the probability of observing less than *10 *predators in the first *100 *days of simulation?"*. Similar questions become immediate once a graphical representation of the involved probabilities is available and, in a model-refinement loop as the one depicted in Figure 1, might lead to further questions such as *"Can we find parameter values which guarantee that the probability of observing less than *10 *predators is less than *.001*?"*.

For availability and requirements, see Table 2.

**Table 2:** 

Availability and requirements	
**Project name:**	PYTSA (Python Time-series Analyzer)
**Version:**	0.3.8
**Homepage:**	http://bimib.disco.unimib.it/
**Operating systems:**	platform independent
**Programming language:**	Python
**Requirements:**	Python (≥ v. 2.7), NUMPY (≥ v. 1.6.1),
	SCIPY (≥ v. 0.10.1), MATPLOTLIB (≥ v. 1.3.0),
	PANDAS (≥ v. 0.12.0) and PYTABLES (≥ v. 2.3.1).
**License:**	BSD 3-clause ("BSD new", 1999)

## Conclusions

In the light of automatising the common approaches to the analysis of models of biological systems, we introduced the reader to PYTSA, a novel Python Time-Series Analyser to analyse synthetic/wet biological time-course simulation experiments. This lightweight, simulation-framework-independent and focused library combines several analyses, with intuitive commands, which can be pipelined with any simulation tool, allowing to generate reports in a very intuitive way. Also, PYTSA supports SBRML to load/export analysis results in a format likely to become a standard, possibly allowing the tool to be pipelined further.

Despite being in its infancy, this tool sets the basis of a novel open source data-analysis framework for the community. PYTSA relies on standard Python APIsforscientific computing to provide an off-the-shelf reusable component for the analysis of time-series, that fits between integrated simulation/analysis environments and "general" tools like Matlab and Mathematica. Its future implementations might enlarge the class of supported input time-series to account, for instance, for spatial models [31] which are generally characterised by more complex output types. Similarly, web services might be implemented for remote-analysis capabilities, and support of data-exchange standard formats beyond SBRML will be considered, so to fit PYTSA within the"ecosystem" established by ongoing standardisation efforts [29,30].

## Competing interests

The authors declare that they have no competing interests.

## Authors' contributions

GC and MA conceived this study, GC and LDS engineered the tool, LDS implemented it and performed the tests, GC wrote the manuscript, which all the authors revised.

## Supplementary Material

Additional file 1 **Supplementary Material Supplementary description of the prey-predator model, of the parameters used for its simulation and further analysis are shown**. Click here for file

## Declarations

Publications costs for this article were funded by Dipartimento di Informatica, Sistemistica e Comunicazione, Università degli Studi di Milano-Bicocca, Italy.

This article has been published as part of *BMC Bioinformatics *Volume 16 Supplement 9, 2015: Proceedings of the Italian Society of Bioinformatics (BITS): Annual Meeting 2014: Bioinformatics. The full contents of the supplement are available online at http://www.biomedcentral.com/bmcbioinformatics/supplements/16/S9.
