# Supplementary material for: Automatising the analysis of stochastic biochemical time-series
Source: BMC Bioinformatics. 2015 Jun 1;16(Suppl 9):S8. doi: 10.1186/1471-2105-16-S9-S8 (PMC4464019; doi:10.1186/1471-2105-16-S9-S8)
Supplement: Additional file 1 — Supplementary Material Supplementary description of the prey-predator model, of the parameters used for its simulation and further analysis are shown. [file 1471-2105-16-S9-S8-S1.pdf]

Automatising the analysis of stochastic biochemical time-series: supplementary material

Giulio Caravagna* Luca De Sano Marco Antoniotti

Dipartimento di Informatica, Sistemistica e Comunicazione,
Università degli Studi di Milano-Bicocca,
Milano, Italy.

September 17, 2014

We provide a detailed discussion of the model presented in the main text, and its analysis with pyTSA. For an exhaustive general introduction of prey-predators models we refer the reader to [1].

Model specification

We denote with $Y_1(t)$ and $Y_2(t)$ preys and predators at time t, respectively, we model that:

(i) preys reproduce and die at rates α and α/γ, respectively;

(ii) predators eat preys at rate β;

(iii) predators die at rate δ.

A mean-field deterministic representation of the model is constituted by the following set of coupled *differential equations*

$$\frac{dY_1}{dt} = \alpha Y_1 \left(1 - \frac{Y_1}{\gamma}\right) - \beta Y_1 Y_2 \qquad \frac{dY_2}{dt} = \beta Y_1 Y_2 - \delta Y_2 \,. \tag{1}$$

In this case the growth of preys is logistic, $\alpha Y_1(1-Y_1/\gamma)$, meaning that the preys grow within an environment with a carrying capacity (or plateau) proportional to γ. Term δY_2 models the death of the predators. The eating of preys by predators is given by the non-linear term $\beta Y_1 Y_2$; such a process, which increases (resp. decrease) the predators population (resp. preys), abstracts the evolutionary cycle "hunt-eat-reproduce" of each individual.

It is possible to represent this model as a non-linear birth-death stochastic process, and simulate it with the standard Gillespie algorithm [2]. In this case it is convenient to use the following reaction notation:

$$(\texttt{growth}-\texttt{up}) \quad Y_1 \overset{\alpha}{\longmapsto} 2Y_1 \qquad (\texttt{growth}-\texttt{down}) \quad 2Y_1 \overset{\alpha/\gamma}{\longmapsto} Y_1$$

$$(\texttt{predation}) \quad Y_1 + Y_2 \overset{\beta}{\longmapsto} 2Y_2 \qquad (\texttt{death}) \quad Y_2 \overset{\delta}{\longmapsto} \emptyset$$

which is an equivalent representation of model (1). In the area of stochastic modelling this representation is often written algebraically with its *stoichiometry matrix* and the set of *propensity functions*. These are

$$D = \begin{bmatrix} 1 & -1 & -1 & 0 \\ 0 & 0 & 1 & -1 \end{bmatrix} .$$

*Corresponding author: giulio.caravagna@disco.unimib.it

and

$$a_1(Y_1, Y_2) = \alpha Y_1 \qquad a_2(Y_1, Y_2) = \alpha Y_1(Y_1 - 1)/2\gamma$$
$$a_3(Y_1, Y_2) = \beta Y_1 Y_2 \qquad a_4(Y_1, Y_2) = \delta Y_2\,.$$

The dataset analysed in the main text is generated by simulating the above stochastic model with NoisySIM [3] with parameters (time units are *days*)

$$\alpha = 0.1 \qquad \gamma = 100$$
$$\beta = 0.001 \qquad \delta = 0.1\,.$$

The dataset consists of 100 independent simulations evaluated by independent NoisySIM runs starting from the initial number of 100 preys and 100 predators. Each simulation is represented by NoisySIM as a `csv` (comma separated values) file.

Dataset analysis with pyTSA

We show and comment, line-by-line, the result of executing a slightly extended version of the pyTSA's script presented in the main text, with the input dataset described above. For explanatory purposes, we show the kind of questions one might raise when analysing data with pyTSA.

To start using the tool is sufficient to use a standard python environment and load the library with the following command

```
> import pytsa as tsa
```

The output returned to console by processing the script is omitted.

How do I load my data?

pyTSA assumes that

- every model simulation is stored in a separate `csv`/`tsv`/SBRML/... file;
- if the files is `csv`, as it is the case here, each column is a variable of the model, so in this case one column is time, the free-variable, and two others are the time-evolution of preys and predators.

These are the basic assumptions to run the tool, and further options for data-loading are explained in detail in the pyTSA manual. The input files, in this case, look like the following

File 1		
t	Y_1	Y_2
0	100	100
0.01	96	97.5
0.04	92	100.5
......		

File 2		
t	Y_1	Y_2
0	100	100
0.1	99	97
0.189	94	92
......		

Notice that there is no requirements that the time-series are discretised, in time, with the same granularity. pyTSA will take care of sampling uniformly from each input time-series.

The input dataset can be loaded (we assume the dataset to be stored in the current folder), and mnemonic names `time` `Preys` and `Predators` can be assigned to the columns. In this case we are loading a simple set of `.csv` file, so no other options are required.

```
> mydata = tsa.dataset('.', colnames=['time', 'Preys', 'Predators'])
```

Once data is loaded, pyTSA can be set to export the plots we show below in different types of formats (`csv`/`tsv`/SBRML/..., as discussed in the manual (not shown here).

Can I view the set of traces in my dataset?

Of course you can plot the data as-it-is. In particular one cane plot all the time series, as a function of time, and as 2D/3D phase spaces. To optimise visualisation, plots can be restricted to consider only a subset of the loaded columns or, for instance, a small time-interval.

For the sake of readability, in the following plot we show a subset of the input datasets (obtained by loading only a small percentage of the 100 input files), we restrict to time $t \leq 1000$, for the plain time-series, and to $t < 100$ for the state-space, and we plot the preys/predators in two separate panels (via flag `merge`).

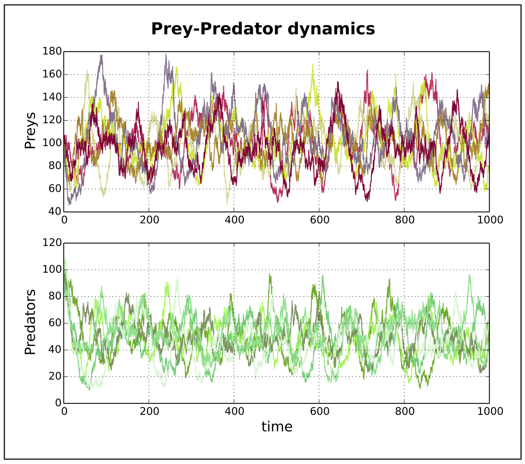

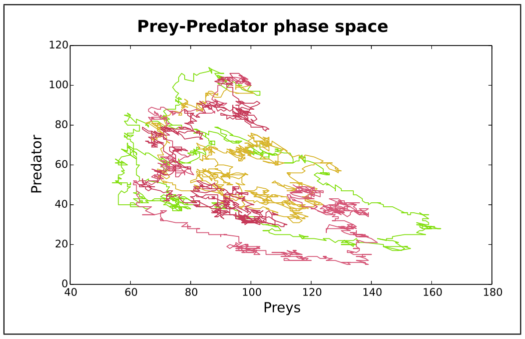

The above plots are obtained by executing the following commands

```
> mydata.splot(columns=['Preys','Predators'], stop=1000, merge=False)
> mydata.phspace(columns=['Preys','Predators'], stop=100)
```

These plots suggest an oscillatory behaviour predicted by the model, which can be further investigated with PyTSA.

<u>*Which is the behaviour of the average number of preys/predators?*</u>

By averaging, over time, many time-series PyTSA evaluates the *expectation* $\mathbb{E}[\cdot]$ of a system variable Y_i, as well as its *standard deviation* μ_{Y_i} at time t. These measures can be plot with bars or lines, restricted to some columns or time interval and evaluated in the phase space, as shown below.

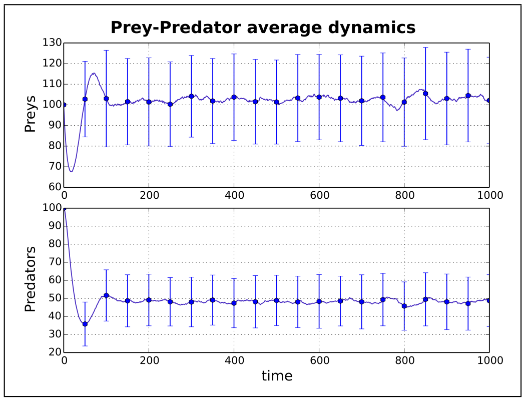

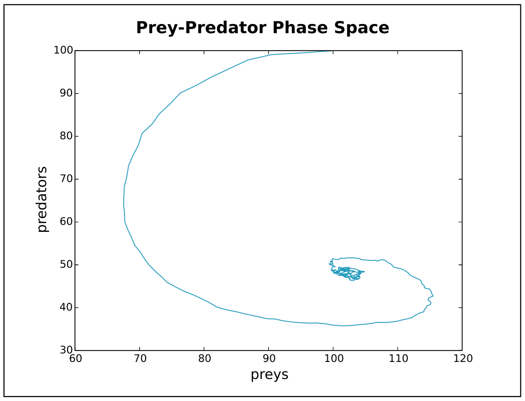

The above plots are obtained by executing the following commands

```
> mydata.asdplot(columns=['Preys','Predators'], stop=1000, merge=False, errorbars=True, numbars=20)
> mydata.aphspace(columns=['Preys','Predators'], stop=100)
```

These plots suggest that the average number of preys doubles the average number of predators. This might suggest that, in general, predators oscillate - with high probability - within lower values than preys. This fact can be investigated by using the empirical estimation of the prey/predator probabilities with PyTSA.

What is the probability *of finding "k" preys/predators over time?*

Since the model was repeatedly simulated from its initial state PyTSA can estimate (empirically) the *probability distribution* for each system variable Y_i to take some value k, at a specific time-point or for a whole time-interval. In a stochastic model as the one described here, this is equivalent to estimating - numerically - the solution of the master equation of the system.

For instance, we might want to assess, at $t = 100$, the probability distribution of the number of preys and predators, and we might want to fit it (if unimodal) with a Gaussian distribution. Also, we might be interested in assessing the same kind of information for preys at various time-points, say $t \in \{25, 50, 100\}$. Such distributions look like the following

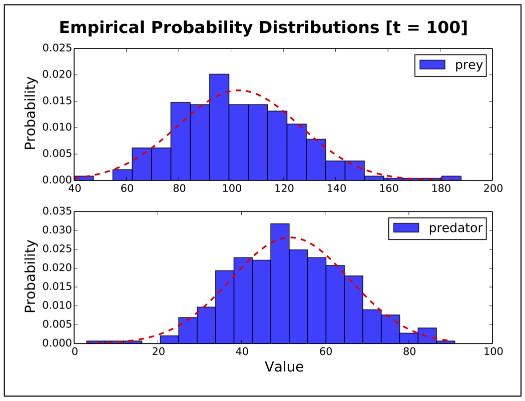

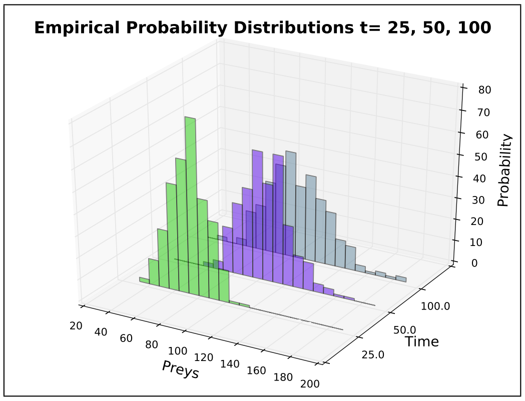

and are obtained by executing the following commands

```
> mydata.pdf(100,  merge=False, fit=True)
> mydata.pdf3d('Preys', [25,50,100])
```

Results of the Gaussian fit (μ and σ^2) for the left plot are returned, via console, by PyTSA.

Now, by looking at these distributions it should be evident that after 100 days the number of preys and predators seems robust, meaning that the probability of any Y_i to reach 0 seems 0. However, we might be interested in understanding whether and how the system oscillates, over-time, and if it reaches any critical boundary region with, e.g., a very low number of individuals. Assessing this particular fact is of special interest in this case since, even if model seems to forecast sustainability, unpredictable events (e.g., an epidemic or a drought, which are not explicitly accounted for within the model equations) happening when the species are in the critical regions might lead to individuals' extinction.

In PyTSA you can evaluate a time-varying probability distribution (heatmap or 3D surface) to answer such questions. For instance, we show such a distribution in the interval $0 \leq t \leq 100$, as heatmap (in two separate panels for both species), and for `Preys` as 3D surface plot.

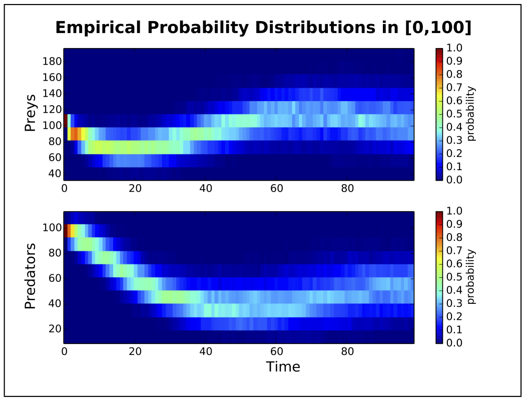

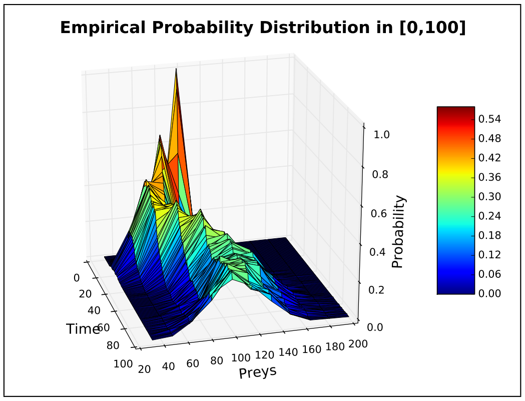

These two plots are obtained by executing the following commands

```
> mydata.meq2d(100,  merge=False, start=0, stop=100)
> mydata.meq3d('Preys', start=0, stop=100)
```

For instance, by analysing the above plots it can be observed that the system oscillates with period around 100 and that the number of predators reaches a critical region ($Y_2 < 20$) with non-negligible probability.

References

[1] JD Murray, Mathematical Biology: I. An Introduction (551 pages), 2002; Mathematical Biology: II. Spatial Models and Biomedical Applications (811 pages), 2003.

[2] DT Gillespie, A General Method for Numerically Simulating the Stochastic Time Evolution of Coupled Chemical Reactions. Journal of Computational Physics 22 (4): 403-434, 1976.

[3] G Caravagna, G Mauri and A d'Onofrio, NoisySIM: exact simulation of stochastic chemically reacting systems with extrinsic noises. Proc. of the Symposium on Theory of Modeling and Simulation, Society for Computer Simulation International 12, 2013.
